# Evaluation of 18 quality indicators from the external quality assurance preanalytical programme of the Spanish Society of Laboratory Medicine (SEQC^ML^)

**DOI:** 10.1515/almed-2021-0097

**Published:** 2022-05-18

**Authors:** Andrea Caballero, Rubén Gómez-Rioja, Montserrat Ventura, María Antonia Llopis, Josep Miquel Bauça, Carolina Gómez-Gómez, Itziar Marzana, Mercedes Ibarz

**Affiliations:** Extra-analytical Quality Commission of the Spanish Society of Laboratory Medicine (SEQC^ML^). Department of Clinical Biochemistry, Echevarne Laboratory, Sant Cugat del Vallés, Spain; Extra-analytical Quality Commission of the Spanish Society of Laboratory Medicine (SEQC^ML^). Servicio de Análisis Clínicos. Hospital La Paz-Cantoblanco-Carlos III, Madrid, Spain; Extra-analytical Quality Commission of the Spanish Society of Laboratory Medicine (SEQC^ML^). External Quality Assurance Programmes, Spanish Society of Laboratory Medicine, Barcelona, Spain; Extra-analytical Quality Commission of the Spanish Society of Laboratory Medicine (SEQC^ML^). Clinical Laboratories Corporate Manager, Catalan Institute of Health (ICS), Barcelona, Spain; Extra-analytical Quality Commission of the Spanish Society of Laboratory Medicine (SEQC^ML^). Servei d’Anàlisis Clíniques, Hospital Universitari Son Espases, Palma, Spain; Extra-analytical Quality Commission of the Spanish Society of Laboratory Medicine (SEQC^ML^). Department of Clinical Laboratory, University Hospital Germans Trias I Pujol, Badalona, Barcelona, Spain; Extra-analytical Quality Commission of the Spanish Society of Laboratory Medicine (SEQC^ML^). Unidad extraanalítica, Laboratorios Hospital Universitario Cruces, Baracaldo, Vizcaya, Spain; Extra-analytical Quality Commission of the Spanish Society of Laboratory Medicine (SEQC^ML^). Department of Clinical Laboratory, University Hospital Arnau de Vilanova, IRBLleida, Lleida, Spain

**Keywords:** external quality assurance programmes, performance specifications, preanalytical phase, quality indicators

## Abstract

**Objectives:**

Most errors in laboratory medicine occur in the pre- and post-analytical phases of the total testing process (TTP). In 2014, the Spanish Society of Laboratory Medicine (SEQC^ML^) started the current Preanalytical Phase EQA Programme, with the objective of providing a tool for the improvement of the preanalytical phase. The aim of this study was to review the evolution of quality indicators (QI) and the comparability of established performance specifications (PS) with other EQA programmes.

**Methods:**

In the SEQC^ML^ programme, participants were asked to register rejections of the main specimens and the causes for rejections. Data collected from 2014 to 2017, and then reviewed biennially (2018–2019), was used to calculate the percentiles; p25, p50, p75, and p90 for every round, and their means were set as PS. These PS were compared with the results of other programmes.

**Results:**

The evolution of QI results for 2018–2019 period showed general maintenance or improvement, e.g., a significant decrease in the number of serum samples with a haemolytic index ≥0.5 g/L, except for EDTA and citrate samples handle, maybe for an improvement in detection. The comparison with PS for the QI of the IFCC Working Group “Laboratory Errors and Patient Safety” and the Key Incident Management and Monitoring System (KIMMS) programme of the RCPA showed comparable results, supporting the validity of the established specifications.

**Conclusions:**

The PS obtained are a helpful tool for benchmarking and to identify processes of the preanalytical phase whose improvement should be set as a priority.

## Introduction

A substantial body of evidence has demonstrated that most errors in laboratory medicine occur in the pre- and post-analytical phases of the total testing process (TTP) which, in turn, translate into a higher risk for the patient [1]. Although the concept of the brain-to-brain laboratory loop was described more than 45 years ago [2], awareness and consensus on the importance of extra-analytical aspects in laboratory quality are a more recent achievement [3, 4]. All steps of the TTP should be continuously monitored and evaluated to ensure that high quality laboratory results are provided [5].

The establishment of internal quality monitoring systems [6] and the participation in external quality assurance (EQA) programmes represent essential tools for the continuous improvement of these processes [7, 8]. Quality indicators (QI), managed as a part of laboratory improvement strategy, have proven to be a suitable tool in monitoring and improving performance in the extra-analytical phase [9, 10]. In fact, according to the ISO 15189:2012, the identification and use of effective QIs in all phases of the TTP is an essential requirement for laboratory accreditation [8], ideally checking the entire examination process, including pre and post-examination procedures [11].

In the last decades, three different types of EQA schemes for the preanalytical phase have been developed in some countries (Table 1) [12]: type I; registration of procedures, type II; circulation of samples simulating errors, and type III; registration of errors/adverse events. In this latter case, the EQA organizer should also lead the harmonization of QIs for a valid comparison of error rates and provide performance specifications (PS). Three of the programmes included in this type have established PS based on the state of the art; the International Federation of Clinical Chemistry and Laboratory Medicine (IFCC) [13], the Royal College of Pathologists of Australasia (RCPA) [14], and the Spanish Society of Laboratory Medicine (SEQC^ML^) programme.

**Table 1:** EQA programmes for the extra-analytical phase (modified from Kristensen et al. 2014 [12]).

Type	Programme	Organization	Country	Starting year	Web site
**Type I**	Preanalytical phase EQA programme	SEQC^ML^	Spain	2001^a^	
	NOKLUS preanalytical EQAs	NOKLUS^b^	Norway	2008	http://www.noklus.no
	Four preanalytical EQAs programmes (online training)	INSTAND e.V	Germany	2011	https://www.instand-ev.de/en
	Preanalytics programmes in clinical chemistry, anatomic pathology, microbiology, urine, and blood sample collection and POCT^c^	Labquality	Finland	2014	https://www.labquality.fi/en/eqas/?lang=en
**Type II**	Preanalytical serum indices scheme	WEQAS^d^	UK	2010	http://www.weqas.com
	EQA programme for serum indices	SEQC^ML^	Spain	2018	https://www.seqc.es/es/programas-garantia-calidad
	EQA programme addressing the important preanalytical workflows applied to personalized medicine	SPIDIA^e^ and SPIDIA4P	Partners from Denmark, UK, Switzerland, Sweden, Italy, Austria, Luxembourg, France, Netherlands, and Spain	2011	https://www.spidia.eu
**Type III**	The Q-track programmes	CAP^f^	USA	1998	https://www.cap.org/laboratory-improvement/quality-management-Programmes
	Key incident monitoring and management systems quality assurance (KIMMS)	RCPA	Australia	2009	https://rcpaqap.com.au
	Preanalytical phase EQA programme	SEQC^ML^	Spain	2014	https://www.seqc.es/es/programas-garantia-calidad
	NOKLUS preanalytical EQAs	NOKLUS	Norway	2017	http://www.noklus.no
	UK NEQAS pre- and post-analytical quality monitoring service	UK NEQAS^g^	United Kingdom	2019	https://birminghamquality.org.uk/prepq/
	Model of quality indicators of WG-LEPS	IFCC	Worldwide	2017	https://www.ifcc.org/ifcc-education-division/working-groups-special-projects/laboratory-errors-and-patient-safety-wg-leps/quality-indicators-project/

^a^Finished in 2013. ^b^Norwegian Organization for Quality Improvement of Laboratory Examinations, ^c^Point-of-care testing, ^d^Wales External Quality Assurance Scheme, ^e^Standardization and Improvement of Generic Pre-analytical Tools and Procedures for *In Vitro* Diagnostics, ^f^College of American Pathologists, ^g^United Kingdom National External Quality Assessment Service.

A PS is a quantitative value that requires implementation of corrective measures when it is exceeded [15] and in line with the claims of the first Strategic Conference of the European Federation of Clinical Chemistry and Laboratory Medicine (EFLM) held in Milan in 2015, the definition of PS for the extra-analytical phase should follow the same hierarchical models as those for the analytical PS: clinical outcome, biological variability, and state of the art [9, 15–18]. Nevertheless, in this phase of the TTP, the state of the art is the most feasible criterion to be readily applicable [1, 10]. Three levels of PS were also suggested to be defined, in accordance with the model developed by Fraser et al. for analytical specifications [19]: first level: individual results below the 25th percentile (p25) of value distribution, representing the best performance; second level: 50th percentile (p50) value, representing the more frequent/common performance, and third level: 75th percentile (p75), representing the worst performance [17]. Other levels might be also used, such as the 10th percentile (p10) to designate “first class laboratories”; the majority of laboratories being grouped between the p10 and 90th percentile (p90); and those with PS values outside the p90, being considered as having the “worst performance”, or alternatively, a one limit model set at the p25 to define the acceptable or unacceptable performance [20].

In 1998 the Commission on Extra-analytical Quality of the SEQC^ML^ started a pilot type I programme for the quality assurance of the preanalytical phase, which was later consolidated in 2001. Until 2013, the programme focused on the analysis of the causes of blood and urine sample rejection. The results for the first five years of the Programme (2001–2005) were published in 2006 for urine [21] and in 2008 for blood samples [22], while an evaluation of the whole 13 year period (2001–2013) was published in 2016 [7]. This programme provided information on the main modes of error in the preanalytical process in Spanish laboratories, which were haemolysis, followed by non-received and clotted for blood samples, and non-received for urine samples.

In 2014 the programme was redesigned as type III to improve user accessibility, thus enabling easier data collection and submission, and to enhance robustness by increasing the length of time for each laboratory to register rejections. Since then, data from participating laboratories has been processed and analysed, and p25, p50, p75, and p90 have been used as PS. The main objective of the programme is to offer an easy tool for the detection of the most frequent errors in the preanalytical phase, as well as the promotion of cross-comparison of results and, thus, stimulate continuous improvement [23]. Each laboratory is encouraged to implement corrective measures when the results of their QI exceed the proposed PS [15].

The aim of this study was to review the consistency and evolution of QI, first stablished in a 4 year period (2014–2017) and then reviewed biennially (2018–2019), and assess the comparability of established PS with the EQA programmes of the Working Group “Laboratory Errors and Patient Safety” (WG-LEPS) of the IFCC and the Key Incident Management and Monitoring System (KIMMS) Programme of the RCPA.

## Materials and methods

### Programme design

In the SEQC^ML^ preanalytical programme, participants are asked to register rejections and their causes for the main specimens observed for one month, four times per year. Then, QIs are calculated as percentage of rejections respect to the total number of the most frequently measured test in each type of sample (creatinine for serum, complete blood count for whole-blood EDTA, and prothrombin time for plasma citrate samples), although in some cases they are referred to the total number of requests when there is not a main type of specimen or test performed. QI definitions and the mean value of p25, p50, p75, and p90 used to calculate PS are described in Table 2.

**Table 2:** List of quality indicators (QI) of the SEQC^ML^ preanalytical programme and their formula for calculation. Performance specifications (PS) calculated from the results of the programme from 2014 to 2017 and from 2018 to 2019, median difference between them (negative when there is an improvement in p50 and positive when there is a worsening) are shown.

Type of QI	QI	Formula	PS 2014–2017				PS 2018–2019				Median difference
			p25	p50	p75	p90	p25	p50	p75	p90	
General rejections	PRE-01	Total rejections/Total requests (%)	1.409	2.266	3.272	4.695	1.393	2.182	3.215	4.608	−0.084
	PRE-02	Unlabelled samples/Total requests (%)	0.000	0.009	0.041	0.086	0.000	0.014	0.049	0.102	0.005
	PRE-03	Misidentified samples/Total requests (%)	0.001	0.010	0.030	0.068	0.000	0.009	0.020	0.045	−0.001
Serum sample rejections	PRE-04	Total serum sample rejections/Creatinine tests (%)	0.452	1.091	2.224	3.534	0.418	0.853	1.737	3.696	**−0.238** ^a^
	PRE-05	Not received serum samples/Creatinine tests (%)	0.102	0.188	0.328	0.678	0.104	0.210	0.350	0.616	0.022
	PRE-06	Haemolysed serum samples/Creatinine tests (%)	0.222	0.718	1.791	2.876	0.146	0.483	1.397	3.365	**−0.235** ^a^
	PRE-07	Insufficient serum samples/Creatinine tests (%)	0.004	0.037	0.107	0.232	0.003	0.035	0.111	0.252	−0.002
Whole-blood EDTA sample rejections	PRE-08	Total whole-blood EDTA sample rejections/Complete blood counts (%)	0.288	0.481	0.766	1.143	0.317	0.519	0.769	1.066	**0.038** ^a^
	PRE-09	Not received whole-blood EDTA samples/Complete blood counts (%)	0.134	0.248	0.414	0.751	0.167	0.266	0.401	0.624	0.018
	PRE-10	Insufficient whole-blood EDTA samples/Complete blood counts (%)	0.005	0.026	0.068	0.146	0.005	0.022	0.062	0.113	−0.004
	PRE-11	Clotted whole-blood EDTA samples/Complete blood counts (%)	0.072	0.150	0.252	0.443	0.087	0.162	0.288	0.456	0.012
Plasma coagulation citrate rejections	PRE-12	Total plasma coagulation citrate sample rejections/Prothrombin tests (%)	0.811	1.748	2.863	4.734	0.937	1.887	3.298	5.471	0.139
	PRE-13	Not received plasma coagulation citrate samples/Prothrombin tests (%)	0.371	0.833	1.565	2.273	0.388	0.743	1.468	2.441	−0.090
	PRE-14	Insufficient plasma coagulation citrate samples/Prothrombin tests (%)	0.112	0.430	1.028	2.028	0.150	0.465	1.230	2.629	0.035
	PRE-15	Clotted plasma coagulation citrate samples/Prothrombin tests (%)	0.015	0.146	0.381	0.822	0.042	0.204	0.474	0.920	0.058
	PRE-16	Haemolysed plasma coagulation citrate samples/Prothrombin tests (%)	0.000	0.000	0.077	0.384	0.000	0.006	0.095	0.557	**0.006** ^a^
Urine sample rejection	PRE-17	Not received urine samples/Total requests (%)	0.351	0.804	1.359	2.017	0.393	0.877	1.370	1.869	0.073
Sample quality	PRE-18	Serum samples with haemolytic index ≥0.5 g/L/Creatinine tests (%)	0.852	1.861	3.399	5.400	0.758	1.503	2.859	4.988	**−0.358** ^a^

^a^Statistical significance in median test (p<0.05).

### Definition of a rejection

A rejection was considered when one or several results from a request cannot be delivered due to preanalytical errors (a test is not performed or reported, as the specimen does not meet laboratory acceptability criteria). The rejection may be global, invalidating all the tests requested, or it may affect a specific test, allowing the rest to be reported.

In laboratory information systems (LIS), tests are usually grouped into requests or orders. In some systems, the request must correspond to a single type of specimen/sample, although in Spain it is usual for LIS to allow each order to contain tests belonging to different specimens. For this reason, the programme requires that rejections are collected according to the sample in which the determination has been made, i.e., the rejected test refers to the type of specimen/container from which it originates. The four types included in the programme are the most common specimens in the laboratory: serum (as in Spanish routine laboratories it is more common to use serum than plasma for biochemistry tests, and usually with separating gel [24]), whole blood EDTA, plasma citrate, and random urine. Table 2 shows the calculation formula (numerator and denominator) for the different QIs.

### Performance specification

**Figure 1:**
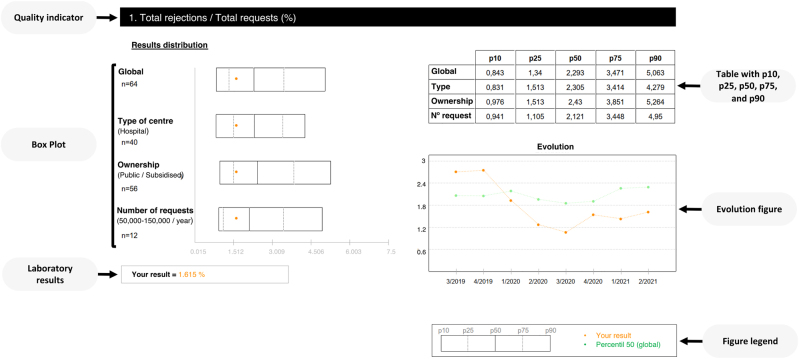
Example of the results reports for indicator number one (PRE-01) of the pre-analytical programme of SEQC^ML^. Four box plots are shown, in which the result of the laboratory indicator is compared with: (1) global; the percentiles calculated globally for all the laboratories, (2) type of centre; the percentiles calculated for the same type of centre (hospital, hospital + primary care, or independent laboratory), (3) ownership; the percentiles calculated for the same type of laboratory ownership (private or public/subsidised), (4) number of request; percentiles calculated for laboratories with the same number of request (25,000–50,000/year, 50,000–150,000/year, 150,000–300,000/year, 300,000–600,000/year, or >600,000/year).

All data collected from each participant´s submission (four times per year) were processed and the results of each laboratory are compared with respect to the p25, p50, p75, and p90 derived from the distribution of all results of the round. A p50 skew graph is additionally provided (Figure 1).

PS of the programme, calculated for the first time using 2014–2017 data, were recalculated on a biennial basis, the percentiles mean of eight submissions are used for the establishment of PS as follows: p25 as the optimum specification, p50 as the desirable specification, p75 as the minimum specification, and also p90, because it is considered that voluntarily participating in an EQA programme and remaining in the central 90% of the distribution of results can be considered a reasonable performance, in a similar way as in Biochemistry programmes where there are no higher-level specifications. The results obtained were compared to the previously established period as median variation (median test), if significant improvement was noticed, new specifications were adopted.

### Data analysis

IBM SPSS v21 software was used for all statistical analyses (median test). Statistical significance was set at 0.05.

## Results

**Figure 2:**
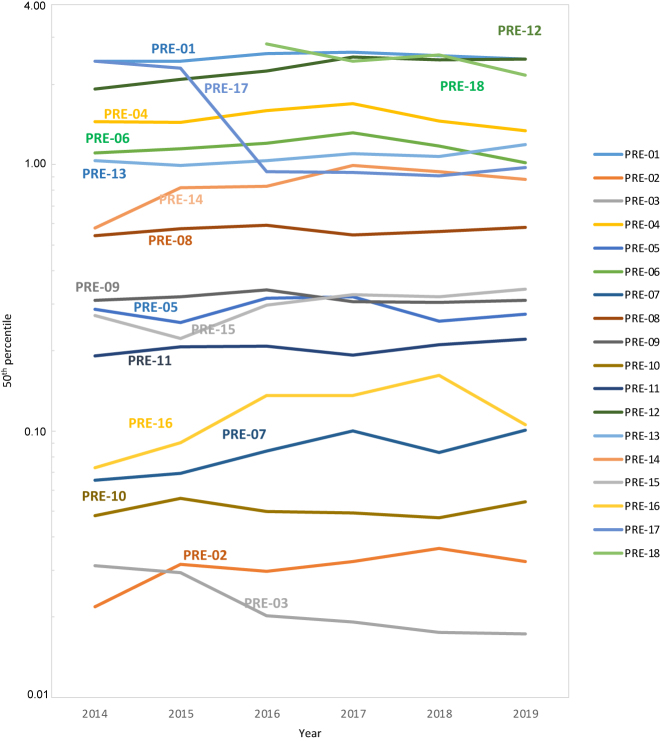
Mean for annual values of the p50 for each of the quality indicators (QIs) from 2014 to 2019.

A total of 63 laboratories participated in the first period between 2014 and 2017, while 72 laboratories participated in the two-year period 2018–2019. PS for both periods are shown in Table 2. A decrease was seen in PRE-04: Total serum sample rejection, PRE-06: Haemolysed serum samples, and PRE-18: Serum samples with haemolytic index ≥0.5 g/L, alongside with an increase in PRE-08: Total whole-blood EDTA sample rejections and PRE-16: Haemolysed plasma coagulation citrate samples (Table 2 and Figure 2). The other QIs show high stability with an inter-period variability below 0.090. For summary data: mean, standard deviation, coefficient of variation, and number of submissions for each percentile (p25, p50, p75, and p90), see Supplementary Material, Table S1.

Table 3 shows the current PS from the programme compared to those established by the IFCC (with 59 participants laboratories) and the KIMMS (with 60 participants laboratories) programmes, as well as coefficient of variation for p50 along the whole period (2014–2019), being comparable with CV from the KIMMS programme. The total the specifications for those indicators that can be matched between programmes, despite the differences in the denominator, showed that the p50 of misidentified samples is lower in the SEQC^ML^ programme compared to KIMMS and IFCC. Nevertheless, the number of misidentified samples was very low in all three (≤0.100). Regarding the total number of samples not received, p50 were higher in KIMMS than those of IFCC and lower than those of SEQC^ML^. However, taking into account the type of specimen, according to the SEQC^ML^ programme, the most frequently not received samples in Spanish laboratories are urine samples (0.877%), followed by plasma coagulation citrate samples (0.743%), whole-blood EDTA samples (0.266%), and serum samples (0.210%). The main cause of rejection of serum samples, haemolysis, showed very similar values to those of IFCC, and lower than in KIMMS. Insufficient and coagulated samples showed the same trend. For CVs, the values were higher in the SEQC^ML^ programme than in KIMMS, showing a greater variability in the laboratories participating in this programme.

**Table 3:** Comparison of the performance specifications (PS) of the SEQC^ML^ programme with those of the QIs of the Working Group “Laboratory Errors and Patient Safety” (WG-LEPS) of the International Federation of Clinical Chemistry and Laboratory Medicine (IFCC) [13] and the Key Incident Management and Monitoring System (KIMMS) of the Royal College of Pathologists Australasia (RCPA) [14].

SEQC^ML^ (2018–2019)						IFCC (2018)					KIMMS (2015–2018)		
QI		p25 (CI 95%)	p50 (CI 95%)	p75 (CI 95%)	CV (%)^a^	QI		p25 (CI 95%)	p50 (CI 95%)	p75 (CI 95%)	QI	Average	CV (%)
PRE-01	Total rejections/Total requests (%)	1.393	2.182	3.215	8.6		NE				NE		
		(1.279–1.506)	(2.102–2.261)	(3.005–3.426)									
PRE-02	Unlabelled samples/Total requests (%)	0.000	0.014	0.049	59.2		NE				Unlabelled	0.084	10
		(0.000–0.000)	(0.010–0.018)	(0.043–0.055)									
PRE-03	Misidentified samples/Total requests (%)	0.000	0.009	0.020	33.8	Pre-MisR	Percentage of: Number of misidentified requests/Total number of requests	0.010	0.025	0.070	Discrepancy of ID	0.100	19
		(0.000–0.001)	(0.006–0.011)	(0.018–0.023)				(0.000–0.010)	(0.020–0.030)	(0.054–0.100)			
PRE-04	Total serum sample rejections/Creatinine tests (%)	0.418	0.853	1.737	16.9		NE				NE		
		(0.395–0.441)	(0.785–0.920)	(1.523–1.951)									
PRE-05	Not received serum samples/Creatinine tests (%)	0.104	0.210	0.350	12.9	Pre-NotRec	Percentage of: Number of samples not received/Total number of samples	0.090	0.190	0.889	Samples not collected	0.317	6
		(0.094–0.114)	(0.199–0.221)	(0.325–0.375)				(0.060–0.100)	(0.140–0.295)	(0.602–1.470)			
PRE-06	Haemolysed serum samples/Creatinine tests (%)	0.146	0.483	1.397	21.2	Pre-HemR	Percentage of: Number of samples rejected due to haemolysis/Total number of checked samples for haemolysis	0.049	0.435	0.882	Haemolysed sample	0.770	7
		(0.103–0.189)	(0.029–0.041)	(1.186–1.607)				(0–0.165)	(0.300–0.500)	(0.587–1.410)			
PRE-07	Insufficient serum samples/Creatinine tests (%)	0.003	0.035	0.111	26.0	Pre-InsV	Percentage of: Number of samples with insufficient volume/Total number of samples	0.010	0.030	0.110	Insufficient sample	0.165	9
		(0.001–0.005)	(0.029–0.041)	(0.099–0.123)				(0.006–0.011)	(0.022–0.040)	(0.079–0.130)			
PRE-08	Total whole-blood EDTA sample rejections/Complete blood counts (%)	0.317	0.519	0.769	12.1		NE				NE		
		(0.298–0.336)	(0.488–0.549)	(0.741–0.797)									
PRE-09	Not received whole-blood EDTA samples/Complete blood counts (%)	0.167	0.266	0.401	7.9	Pre-NotRec	Percentage of: Number of samples not received/Total number of samples	0.090	0.190	0.889	Samples not collected	0.317	6
		(0.155–0.178)	(0.256–0.257)	(0.392–0.410)				(0.060–0.100)	(0.140–0.295)	(0.602–1.470)			
PRE-10	Insufficient whole-blood EDTA samples/Complete blood counts (%)	0.005	0.022	0.062	31.1	Pre-InsV	Percentage of: Number of samples with insufficient sample/Total number of samples	0.010	0.030	0.110	Insufficient sample	0.165	9
		(0.003–0.007)	(0.018–0.026)	(0.057–0.067)				(0.006–0.011)	(0.022–0.040)	(0.079–0.130)			
PRE-11	Clotted whole-blood EDTA samples/Complete blood counts (%)	0.087	0.162	0.288	14.1	Pre-clot	Percentage of: Number of samples clotted/Total number of samples with an anticoagulant checked for clots	0.080	0.237	0.402	Clotted samples	0.193	6
		(0.079–0.094)	(0.148–0.175)	(0.272–0.304)				(0.058–0.111)	(0.200–0.270)	(0.350–0.525)			
PRE-12	Total plasma coagulation citrate imple rejections/Prothrombin tests (%)	0.937	1.887	3.298	10.9		NE				NE		
		(0.842–1.032)	(1.775–1.999)	(2.973–3.623)									
PRE-13	Not received plasma coagulation citrate samples/Prothrombin time (%)	0.388	0.743	1.468	15.1	Pre-NotRec	Percentage of: Number of samples not received/Total number of samples	0.090	0.190	0.889	Samples not collected	0.317	6
		(0.342–0.433)	(0.681–0.805)	(1.348–1–588)				(0.060–0.100)	(0.140–0.295)	(0.602–1.470)			
PRE-14	Insufficient plasma coagulation citrate samples/Prothrombin time (%)	0.150	0.465	1.230	24.7	Pre-SaAnt	Percentage of: Number of samples with inappropriate volume-anticoagulant ratio/Total number of samples with anticoagulant	0.070	0.343	0.770	Incorrect fill of samples	0.089	21
		(0.129–0.172)	(0.430–0.500)	(1.108–1.353)				(0.040–0.114)	(0.220–0.420)	(0.690–0.980)			
PRE-15	Clotted plasma coagulation citrate samples/Prothrombin time (%)	0.042	0.204	0.474	36.8	Pre-clot	Percentage of: Number of samples clotted/Total number of samples with an anticoagulant checked for clots	0.080	0.237	0.402	Clotted samples	0.193	6
		(0.022–0.062)	(0.175–0.232)	(0.441–0.506)				(0.058–0.111)	(0.200–0.270)	(0.350–0.525)			
PRE-16	Haemolysed plasma coagulation citrate samples/Prothrombin time (%)	0.000	0.006	0.095	247.2	Pre-HemR	Percentage of: Number of samples rejected due to haemolysis/Total number of checked samples for haemolysis global	0.049	0.435	0.882	Haemolysed samples	0.770	7
		(0.000–0.000)	(0.000–0.011)	(0.069–0.121)				(0.000–0.165)	(0.300–0.500)	(0.587–1.410)			
PRE-17	Not received urine samples/Total requests (%)	0.393	0.877	1.370	9.0	Pre-NotRec	Percentage of: Number of samples not received/Total number of samples	0.090	0.190	0.889	Samples not collected	0.317	6
		(0.337–0.449)	(0.830–0.923)	(1.325–1.415)				(0.060–0.100)	(0.140–0.295)	(0.602–1.470)			
PRE-18	Serum samples with haemolytic index ≥0.5 g/L/total serum samples where the haemolytic index is performed (%)	0.758	1.503	2.859	15.3	Pre-HemI	Percentage of: Number of samples with free haemoglobin (hb) >0.5 g/L detected by automated haemolytic index/Total number of checked samples for haemolysis	0.690	1.810	3.230	NE		
		(0.686–0.831)	(1.408–1.598)	(2.460–3.259)				(0.460–0.970)	(1.490–2.230)	(2.595–3.800)			

CI 95%, 95% confidence intervals; CV, coefficient of variation; NE, no equivalent indicator. ^a^Coefficient of variation calculated from p50 results from 2014 to 2019.

**Figure 3:**
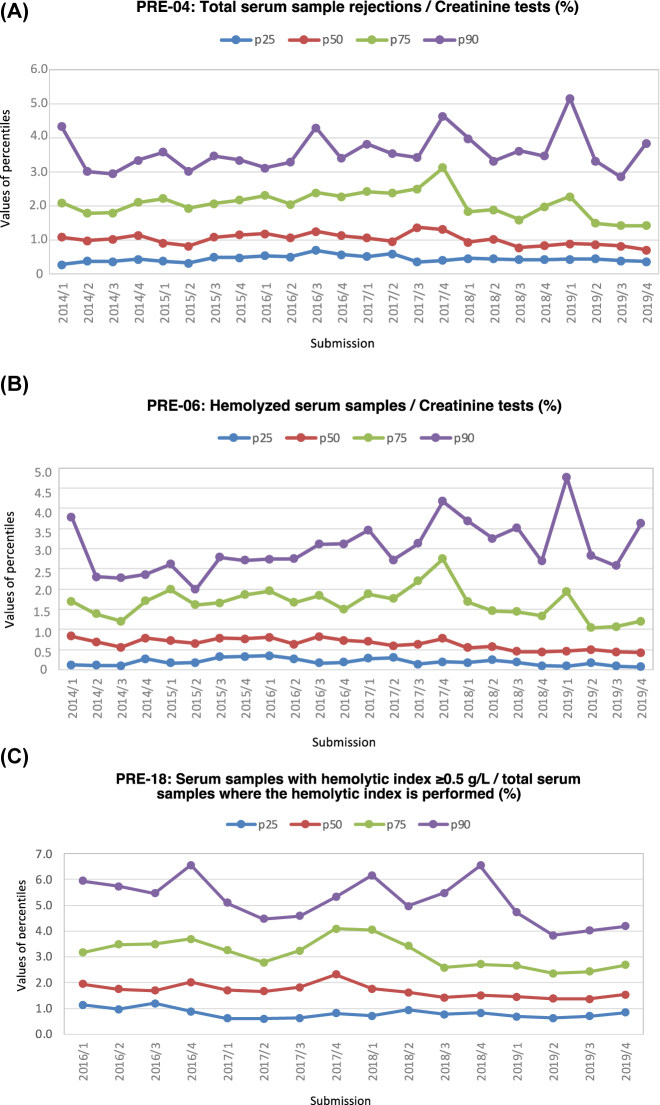
Values from 2014 to 2019 for each of the submissions of the p25, p50, p75, and p90. (A) PRE-04, (B) PRE-06, (C) PRE-18.

A significant decrease in p50 value of total serum sample rejections indicator was observed between periods (Figure 3A), associated with a decrease in the p50 value of rejections due to haemolysis indicator (Figure 3B) (Table 2). The PS was modified to bring it in line with the state of the art of the participants. This indicator was similar to the IFCC *Samples rejected due to haemolysis*. The 95% confidence interval for the median of this indicator in 2018 was 0.06–0.87 [13], although in this case the indicator included all specimens and used as denominator all samples where haemolysis had been measured. In the SEQC^ML^ programme, haemolysed serum (median 0.5%) and citrate (0.006%) samples are reported separately. With respect to the KIMMS programme, the *Haemolysed sample* indicator presents a mean over four years period (2015–2018) of 0.770% with respect to total requests [14]. In both cases, the results are similar to those observed in the SEQC^ML^ programme (Table 3).

The haemolysed serum samples indicator includes rejections, i.e., tests in which at least one result has been removed from the report on the assumption that the result was significantly interfered. Therefore, the prevalence of samples rejected due to haemolysis (mean 0.483%) was lower than the prevalence of samples with a haemolytic index ≥0.5 g/L (1.503%), indicative of sample deterioration (Figure 3C). Serum samples with haemolytic index ≥0.5 g/L indicator were higher than the number of rejections due to haemolysis because low-grade haemolysis can cause interferences that may not be considered significant in the biochemical test results. As with the haemolysis rejection indicator, a significant tendency to decrease was seen and therefore the specifications were modified. This indicator was similar to the IFCC *Samples with free haemoglobin* >0.5 g/L *(automated detection)*, the 95% confidence interval for the median of this indicator in 2018 was 0.67–2.76 [13], using the number of samples in which haemolysis had been measured as the denominator (Table 3). With respect to the KIMMS programme, there no coincident indicator was found.

The number of rejections due to haemolysis in the citrate tube was much lower than in the serum tube (0.006% vs. 0.500%) but an significant increase in the indicator was seen between periods. Total whole blood EDTA sample rejections also showed a significant increase between periods, triggering a change in PS for both indicators. In the case of the whole blood EDTA sample, the main reason for preanalytical rejection was the coagulated sample.

The rest of the indicators showed a very stable evolution with a downward trend. The general indicators stood out, remaining at very low values. PRE-03 misidentified samples/total requests (%), which could be considered a sentinel indicator, had very low values (0.009). This indicator is similar to the *percentage of*: *number of misidentified requests/total number of requests* of the IFCC (0.025%) and the *discrepancy of ID* in KIMMS (0.010%). However, the number of unlabelled samples (PRE-02 unlabelled samples/total requests (%)) increased from 0.009 to 0.014%, this indicator is similar to the KIMMS Unlabelled, which has a higher PS of 0.84%, while no equivalent indicator from the IFCC was found.

## Discussion

There is a general interest in QIs related to the extra-analytical phases, but only a limited number of clinical laboratories collect regular and comprehensive data. The increasing number of participants in the Spanish programme could be considered a reflection of the growing concern in these phases of the TTP, although the number of participants is still far from those enrolled in the programmes of the analytical phase of the SEQC^ML^. To overcome the well-known “quality indicator paradox” [25], the WG-LEPS developed a Model of Quality Indicators (MQI) based on a list of consensually defined QIs (available on www.ifcc-mqi.com) [3, 17, 26] and the collection of data received by clinical laboratories led to the definition of PS in the preanalytical phase [25]. In 2017, the SEQC^ML^ calculated the PS with the results of the past four years collected from the Spanish laboratories, and then recalculated in 2018–2019. The RCPA calculated its own PS based on the results of 2015–2018 period, unlike the other programmes, the results are presented as mean rather than median.

One important drawback of the preanalytical phase quality assurance programmes is the difficulty to compare results due to the different QI formulas applied in their calculations. In this sense, a call for harmonization is needed. According to the results of the SEQC^ML^ programme, QIs calculated with respect to the most frequently measured test in each type of sample gives the most comparable results among Spanish laboratories. Besides, since the number and type of errors in the emergency department (ED) or Intensive Care Units are different and more heterogeneous from that occurred in routine samples [27, 28], the SEQC^ML^ programme includes only data from routine samples to avoid a possible confounding factor in result interpretation. However, some of the indicators are comparable among programmes and show that the PS are very similar for the IFCC and more discrepant with the KIMMS. CVs also show more variation, which could be due to a longer period of years. The SEQC^ML^ programme reflects the stability of the indicators and is therefore useful to see the evolution of each laboratory and whether data is being collected properly.

The SEQC^ML^ programme aims to calculate simple indicators and enables intercomparison by providing an objective reference among different laboratories, establishing PS based on the state of the art. This allows the identification of critical points in the entire preanalytical phase, from the request to the storage and preparation of the sample before analysis, and it is also possible to evaluate the trend over time (positive or negative), since even if the specifications are met, there may be evidence of a deterioration. Stability and/or improvement in the overall indicators (total rejections, unlabelled, and misidentified samples) could reflect the effectiveness of the programme.

The haemolysed sample is by far the main reason for rejection of serum samples. Errors in collection or handling can cause haemolysis of the sample, usually detected in the analytical phase. In fact, in recent years a great effort has been made to improve the process of blood collection, detection of the haemolysed samples, and how to establish measures that help reduce their incidence. The significant decrease observed in serum sample may indicate an improvement in preanalytical practices in participating laboratories and is considered very positive. The minimum specification of the SEQC^ML^ programme suggests that laboratories should keep their number of haemolysis rejections below 1.4% of the total serum samples. This indicator is not far from the 0.9% of the IFCC programme and implies, in any case, that many laboratories reject tests from one out of every 100 samples due to haemolysis.

The percentage of serum samples with haemolytic index ≥0.5 g/L is used as an overall QI of the preanalytical process as it indicates a deterioration of the sample during one of the preanalytical processes (collection, transport, or preparation). The improvement in this indicator could also be considered indicative of awareness of the importance of the preanalytical phase in the laboratory. Also, in this case, results of SEQC^ML^ programme and the IFCC one suggest that laboratories should maintain at least a percentage of haemolysed samples (haemolytic index ≥0.5 g/L) below 3%.

Considering the p75 (minimum specifications) and the type of sample, coagulation citrate samples QIs have the least desirable results and laboratories should take measures to reduce the number of insufficient, clotted, and haemolysed samples. On the other hand, the worsening of the results could reflect the technology improvement of the haemostasis analysers, capable of detecting more preanalytical parameters, in the same way, that happened years ago with serum samples and biochemistry analysers (haemolytic, icterus, and turbidity index). Whatever the reason, is the responsibility of the laboratories to improve QIs. According to the results, the number of clotted whole-blood EDTA samples should also be reduced.

The results between programmes show very similar values in terms of specifications, the CVs are higher compared to KIMMS, but this could be due to the way they are calculated, as in the case of the SEQC^ML^ programme possible outliers are not excluded.

In this study, the results obtained for QI between 2014 and 2017 were used to calculate PS and then compared with those in 2018–2019, to identify processes for which improvement should be a priority. In the next year, a recalculation of the data would be performed, and in the case of better results from the period 2020 to 2021, the specifications may be changed. Nonetheless, the better results generally seen in 2018–2019 reflect the awareness of clinical laboratories in preanalytical phase and the efforts to improve it. In the future, due to the rapid change in diagnostic tests and type of samples in the clinical laboratories (for instance samples for free DNA tests), the validity of the current QIs would need to be revised, and new ones could be included. In this sense, the Extra-analytical Quality Commission of the SEQC^ML^ has included a new QI in the 2021 programme: number of contaminated by infusion serum samples/Creatinine tests (%). In the meantime, the improvement of LIS tools will warrant a simplification in the collection and management of laboratory data, this together with the increase of participants in the EQA scheme, which could also do possible to include ED results.

One of the main limitations of this study is the short number of results, due to the number of participant laboratories (72 in 2018–2019 period), not comparable with the huge volume of data used in the case of analytical phase programmes (around 600 laboratories in 2019 in SERUM EQA Programme, the SEQC^ML^ programme with the highest number of participants), and the not exclusion of outliers’ results. Consequently, the robustness of the PS calculated might be compromised. Another disadvantage of this type of programme, different form the analytical phase schemes, is that in the case of being out of specifications, the measures that must be taken to solve the problem and improve the results of QI, could not be clear. An analysis of the risk associated with each situation should be carried out, this analysis would be useful to prioritize the intervention points. Once the corresponding actions were applied, a comparison of the new QI obtained with the proposed PS would allow verifying the effectiveness of the implemented measures.

In conclusion, preanalytical QIs and PS help identify the existence of errors and assess the effectiveness of implementing corrective actions, but by themselves they do not control anything or identify the causes of non-conformities. It is a shared responsibility between the scientific societies and the individual laboratories to monitor and implement the necessary measures. The SEQC^ML^ preanalytical programme offers a reliable formula to calculate the QIs, and the PS obtained are a helpful tool for benchmarking, as well as to identify processes whose improvement should be a priority for clinical laboratories.

## Supplementary Material

Supplementary Material
