# Supplementary material for: Evaluation of 18 quality indicators from the external quality assurance preanalytical programme of the Spanish Society of Laboratory Medicine (SEQCML)
Source: Adv Lab Med. 2022 May 18;3(2):175–87. doi: 10.1515/almed-2021-0097 (PMC10197339; doi:10.1515/almed-2021-0097)
Supplement: Supplementary file 1 — Supplementary Material [file j_almed-2021-0097_suppl.docx]

**Table S1.** Mean, standard deviation (SD), coefficient of variation (CV), and number of submissions (n) for each percentile (p25, p50, p75, and p90) grouped by time periods, 2019-2014, 2014-2017, and 2018-2019, for each quality indicators (QI). NC: no calculated.

| **p25** | **2014-2019** | | | | **2014-2017** | | | | **2018-2019** | | | |
| --- | --- | --- | --- | --- | --- | --- | --- | --- | --- | --- | --- | --- |
| **QI** | **mean** | **SD** | **CV%** | **n** | **mean** | **SD** | **CV%** | **n** | **mean** | **SD** | **CV%** | **n** |
| PRE-01 | 1.403 | 0.177 | 12.64 | 24 | 1.409 | 0.189 | 13.40 | 16 | 1.393 | 0.164 | 11.77 | 8 |
| PRE-02 | 0.000 | 0.000 | NC | 24 | 0.000 | 0.000 | NC | 16 | 0.000 | 0.000 | NC | 8 |
| PRE-03 | 0.001 | 0.001 | 139.91 | 24 | 0.001 | 0.001 | 119.96 | 16 | 0.000 | 0.001 | 198.41 | 8 |
| PRE-04 | 0.441 | 0.092 | 20.93 | 24 | 0.452 | 0.110 | 24.36 | 16 | 0.418 | 0.033 | 7.98 | 8 |
| PRE-05 | 0.103 | 0.023 | 22.81 | 24 | 0.102 | 0.027 | 26.77 | 16 | 0.104 | 0.014 | 13.69 | 8 |
| PRE-06 | 0.197 | 0.084 | 42.97 | 24 | 0.222 | 0.084 | 37.98 | 16 | 0.146 | 0.062 | 42.30 | 8 |
| PRE-07 | 0.004 | 0.004 | 94.84 | 24 | 0.004 | 0.004 | 99.71 | 16 | 0.003 | 0.003 | 82.20 | 8 |
| PRE-08 | 0.297 | 0.035 | 11.72 | 24 | 0.288 | 0.035 | 12.05 | 16 | 0.317 | 0.028 | 8.69 | 8 |
| PRE-09 | 0.145 | 0.029 | 19.87 | 24 | 0.134 | 0.027 | 20.49 | 16 | 0.167 | 0.017 | 9.98 | 8 |
| PRE-10 | 0.005 | 0.004 | 74.55 | 24 | 0.005 | 0.004 | 81.75 | 16 | 0.005 | 0.003 | 62.34 | 8 |
| PRE-11 | 0.077 | 0.020 | 25.73 | 24 | 0.072 | 0.022 | 29.93 | 16 | 0.087 | 0.011 | 13.12 | 8 |
| PRE-12 | 0.853 | 0.175 | 20.56 | 24 | 0.811 | 0.181 | 22.30 | 16 | 0.937 | 0.137 | 14.65 | 8 |
| PRE-13 | 0.376 | 0.073 | 19.36 | 24 | 0.371 | 0.078 | 20.96 | 16 | 0.388 | 0.065 | 16.89 | 8 |
| PRE-14 | 0.124 | 0.065 | 51.86 | 24 | 0.112 | 0.074 | 65.95 | 16 | 0.150 | 0.031 | 20.47 | 8 |
| PRE-15 | 0.024 | 0.028 | 116.03 | 24 | 0.015 | 0.024 | 155.00 | 16 | 0.042 | 0.029 | 69.29 | 8 |
| PRE-16 | 0.000 | 0.000 | NC | 24 | 0.000 | 0.000 | NC | 16 | 0.000 | 0.000 | NC | 8 |
| PRE-17 | 0.365 | 0.077 | 21.16 | 24 | 0.351 | 0.074 | 21.11 | 16 | 0.393 | 0.081 | 20.50 | 8 |
| PRE-18 | 0.805 | 0.183 | 22.73 | 16 | 0.852 | 0.236 | 27.72 | 8 | 0.758 | 0.105 | 13.79 | 8 |

| **p50** | **2014-2019** | | | | **2014-2017** | | | | **2018-2019** | | | |
| --- | --- | --- | --- | --- | --- | --- | --- | --- | --- | --- | --- | --- |
| **QI** | **mean** | **SD** | **CV%** | **n** | **mean** | **SD** | **CV%** | **n** | **mean** | **SD** | **CV%** | **n** |
| PRE-01 | 2.238 | 0.192 | 8.58 | 24 | 2.266 | 0.219 | 9.66 | 16 | 2.182 | 0.115 | 5.26 | 8 |
| PRE-02 | 0.010 | 0.006 | 59.22 | 24 | 0.009 | 0.006 | 68.46 | 16 | 0.014 | 0.005 | 37.41 | 8 |
| PRE-03 | 0.010 | 0.003 | 33.81 | 24 | 0.010 | 0.003 | 31.50 | 16 | 0.009 | 0.003 | 38.25 | 8 |
| PRE-04 | 1.011 | 0.171 | 16.92 | 24 | 1.091 | 0.143 | 13.07 | 16 | 0.853 | 0.097 | 11.35 | 8 |
| PRE-05 | 0.195 | 0.025 | 12.87 | 24 | 0.188 | 0.026 | 13.81 | 16 | 0.210 | 0.016 | 7.76 | 8 |
| PRE-06 | 0.640 | 0.135 | 21.16 | 24 | 0.718 | 0.083 | 11.60 | 16 | 0.483 | 0.057 | 11.86 | 8 |
| PRE-07 | 0.036 | 0.009 | 25.97 | 24 | 0.037 | 0.010 | 27.27 | 16 | 0.035 | 0.009 | 24.51 | 8 |
| PRE-08 | 0.493 | 0.060 | 12.15 | 24 | 0.481 | 0.064 | 13.27 | 16 | 0.519 | 0.044 | 8.58 | 8 |
| PRE-09 | 0.254 | 0.020 | 7.88 | 24 | 0.248 | 0.020 | 8.24 | 16 | 0.266 | 0.013 | 5.06 | 8 |
| PRE-10 | 0.025 | 0.008 | 31.07 | 24 | 0.026 | 0.008 | 32.39 | 16 | 0.022 | 0.005 | 24.69 | 8 |
| PRE-11 | 0.154 | 0.022 | 14.15 | 24 | 0.150 | 0.022 | 14.93 | 16 | 0.162 | 0.019 | 11.96 | 8 |
| PRE-12 | 1.795 | 0.195 | 10.88 | 24 | 1.748 | 0.198 | 11.35 | 16 | 1.887 | 0.162 | 8.58 | 8 |
| PRE-13 | 0.803 | 0.121 | 15.09 | 24 | 0.833 | 0.126 | 15.14 | 16 | 0.743 | 0.089 | 12.00 | 8 |
| PRE-14 | 0.441 | 0.109 | 24.72 | 24 | 0.430 | 0.129 | 30.01 | 16 | 0.465 | 0.050 | 10.85 | 8 |
| PRE-15 | 0.165 | 0.061 | 36.82 | 24 | 0.146 | 0.061 | 41.71 | 16 | 0.204 | 0.041 | 20.12 | 8 |
| PRE-16 | 0.002 | 0.005 | 247.16 | 24 | 0.000 | 0.001 | 400.00 | 16 | 0.006 | 0.008 | 140.28 | 8 |
| PRE-17 | 0.828 | 0.075 | 9.03 | 24 | 0.804 | 0.068 | 8.48 | 16 | 0.877 | 0.067 | 7.62 | 8 |
| PRE-18 | 1.682 | 0.257 | 15.26 | 16 | 1.861 | 0.222 | 11.94 | 8 | 1.503 | 0.137 | 9.09 | 8 |

| **p75** | **2014-2019** | | | | **2014-2017** | | | | **2018-2019** | | | |
| --- | --- | --- | --- | --- | --- | --- | --- | --- | --- | --- | --- | --- |
| **QI** | **mean** | **SD** | **CV%** | **n** | **mean** | **SD** | **CV%** | **n** | **mean** | **SD** | **CV%** | **n** |
| PRE-01 | 3.253 | 0.325 | 9.98 | 24 | 3.272 | 0.343 | 10.47 | 16 | 3.215 | 0.303 | 9.44 | 8 |
| PRE-02 | 3.253 | 0.325 | 9.98 | 24 | 3.272 | 0.343 | 10.47 | 16 | 3.215 | 0.303 | 9.44 | 8 |
| PRE-03 | 0.044 | 0.011 | 25.55 | 24 | 0.041 | 0.012 | 28.15 | 16 | 0.049 | 0.009 | 18.07 | 8 |
| PRE-04 | 0.027 | 0.011 | 39.76 | 24 | 0.030 | 0.012 | 38.43 | 16 | 0.020 | 0.003 | 16.85 | 8 |
| PRE-05 | 2.062 | 0.389 | 18.85 | 24 | 2.224 | 0.320 | 14.41 | 16 | 1.737 | 0.309 | 17.76 | 8 |
| PRE-06 | 0.335 | 0.040 | 12.01 | 24 | 0.328 | 0.041 | 12.62 | 16 | 0.350 | 0.036 | 10.22 | 8 |
| PRE-07 | 1.659 | 0.379 | 22.83 | 24 | 1.791 | 0.349 | 19.49 | 16 | 1.397 | 0.304 | 21.75 | 8 |
| PRE-08 | 0.108 | 0.020 | 18.64 | 24 | 0.107 | 0.022 | 20.61 | 16 | 0.111 | 0.017 | 15.19 | 8 |
| PRE-09 | 0.767 | 0.086 | 11.24 | 24 | 0.766 | 0.103 | 13.46 | 16 | 0.769 | 0.041 | 5.29 | 8 |
| PRE-10 | 0.410 | 0.040 | 9.88 | 24 | 0.414 | 0.049 | 11.79 | 16 | 0.401 | 0.012 | 3.11 | 8 |
| PRE-11 | 0.066 | 0.010 | 15.85 | 24 | 0.068 | 0.011 | 16.61 | 16 | 0.062 | 0.008 | 12.47 | 8 |
| PRE-12 | 0.264 | 0.038 | 14.26 | 24 | 0.252 | 0.038 | 15.11 | 16 | 0.288 | 0.023 | 8.10 | 8 |
| PRE-13 | 3.008 | 0.415 | 13.81 | 24 | 2.863 | 0.307 | 10.74 | 16 | 3.298 | 0.469 | 14.22 | 8 |
| PRE-14 | 1.533 | 0.135 | 8.80 | 24 | 1.565 | 0.103 | 6.58 | 16 | 1.468 | 0.173 | 11.78 | 8 |
| PRE-15 | 1.096 | 0.209 | 19.07 | 24 | 1.028 | 0.194 | 18.89 | 16 | 1.230 | 0.177 | 14.40 | 8 |
| PRE-16 | 0.412 | 0.089 | 21.67 | 24 | 0.381 | 0.090 | 23.68 | 16 | 0.474 | 0.047 | 9.97 | 8 |
| PRE-17 | 0.083 | 0.042 | 50.17 | 24 | 0.077 | 0.044 | 56.47 | 16 | 0.095 | 0.037 | 39.36 | 8 |
| PRE-18 | 1.363 | 0.070 | 5.13 | 24 | 1.359 | 0.074 | 5.45 | 16 | 1.370 | 0.065 | 4.74 | 8 |

| **p90** | **2014-2019** | | | | **2014-2017** | | | | **2018-2019** | | | |
| --- | --- | --- | --- | --- | --- | --- | --- | --- | --- | --- | --- | --- |
| **QI** | **mean** | **SD** | **CV%** | **n** | **mean** | **SD** | **CV%** | **n** | **mean** | **SD** | **CV%** | **n** |
| PRE-01 | 4.666 | 0.445 | 9.53 | 24 | 4.695 | 0.465 | 9.90 | 16 | 4.608 | 0.426 | 9.25 | 8 |
| PRE-02 | 0.091 | 0.021 | 23.29 | 24 | 0.086 | 0.021 | 24.56 | 16 | 0.102 | 0.018 | 17.66 | 8 |
| PRE-03 | 0.060 | 0.027 | 44.12 | 24 | 0.068 | 0.029 | 43.42 | 16 | 0.045 | 0.007 | 16.44 | 8 |
| PRE-04 | 3.588 | 0.560 | 15.60 | 24 | 3.534 | 0.500 | 14.15 | 16 | 3.696 | 0.688 | 18.61 | 8 |
| PRE-05 | 0.657 | 0.116 | 17.69 | 24 | 0.678 | 0.117 | 17.19 | 16 | 0.616 | 0.111 | 18.04 | 8 |
| PRE-06 | 3.039 | 0.650 | 21.38 | 24 | 2.876 | 0.573 | 19.92 | 16 | 3.365 | 0.708 | 21.05 | 8 |
| PRE-07 | 0.239 | 0.067 | 27.89 | 24 | 0.232 | 0.070 | 30.13 | 16 | 0.252 | 0.061 | 24.43 | 8 |
| PRE-08 | 1.117 | 0.111 | 9.94 | 24 | 1.143 | 0.126 | 11.02 | 16 | 1.066 | 0.045 | 4.25 | 8 |
| PRE-09 | 0.709 | 0.133 | 18.73 | 24 | 0.751 | 0.142 | 18.86 | 16 | 0.624 | 0.049 | 7.90 | 8 |
| PRE-10 | 0.135 | 0.035 | 26.17 | 24 | 0.146 | 0.037 | 25.25 | 16 | 0.113 | 0.018 | 16.36 | 8 |
| PRE-11 | 0.447 | 0.085 | 18.98 | 24 | 0.443 | 0.099 | 22.29 | 16 | 0.456 | 0.052 | 11.31 | 8 |
| PRE-12 | 4.979 | 1.015 | 20.39 | 24 | 4.734 | 0.961 | 20.29 | 16 | 5.471 | 0.998 | 18.24 | 8 |
| PRE-13 | 2.329 | 0.455 | 19.53 | 24 | 2.273 | 0.497 | 21.86 | 16 | 2.441 | 0.359 | 14.70 | 8 |
| PRE-14 | 2.228 | 0.676 | 30.35 | 24 | 2.028 | 0.580 | 28.60 | 16 | 2.629 | 0.712 | 27.06 | 8 |
| PRE-15 | 0.855 | 0.160 | 18.72 | 24 | 0.822 | 0.152 | 18.52 | 16 | 0.920 | 0.165 | 17.94 | 8 |
| PRE-16 | 0.442 | 0.221 | 50.01 | 24 | 0.384 | 0.208 | 54.30 | 16 | 0.557 | 0.210 | 37.74 | 8 |
| PRE-17 | 1.968 | 0.205 | 10.41 | 24 | 2.017 | 0.214 | 10.59 | 16 | 1.869 | 0.152 | 8.15 | 8 |
| PRE-18 | 5.194 | 0.859 | 16.54 | 16 | 5.400 | 0.693 | 12.83 | 8 | 4.988 | 1.003 | 20.10 | 8 |
